# P-1545. Evaluation of Clinical Efficacy of Systemic Antibiotics for Treatment of Acute Pyelonephritis in Adults: A Network Meta-Analysis of Randomized Controlled Trials

**DOI:** 10.1093/ofid/ofae631.1712

**Published:** 2025-01-29

**Authors:** Alireza FakhriRavari, Rita Hanna Al-Kass, Da Young Sohn, Jae Young Sohn, Shant Krikorian

**Affiliations:** Loma Linda University School of Pharmacy, Loma Linda, California; Loma Linda University, Loma Linda, California; Loma Linda University, Loma Linda, California; Loma Linda University, Loma Linda, California; Loma Linda University, Loma Linda, California

## Abstract

**Background:**

Acute pyelonephritis, a severe form of urinary tract infection (UTI), poses significant health risks including kidney damage and urosepsis. Current clinical guidelines, last updated in 2010, primarily focus on women with uncomplicated UTIs, leaving a gap in recommendations for broader cases. Our study aims to bridge this gap by conducting an updated literature review to evaluate the efficacy of various antibiotics for acute pyelonephritis.

**Figure 1:**
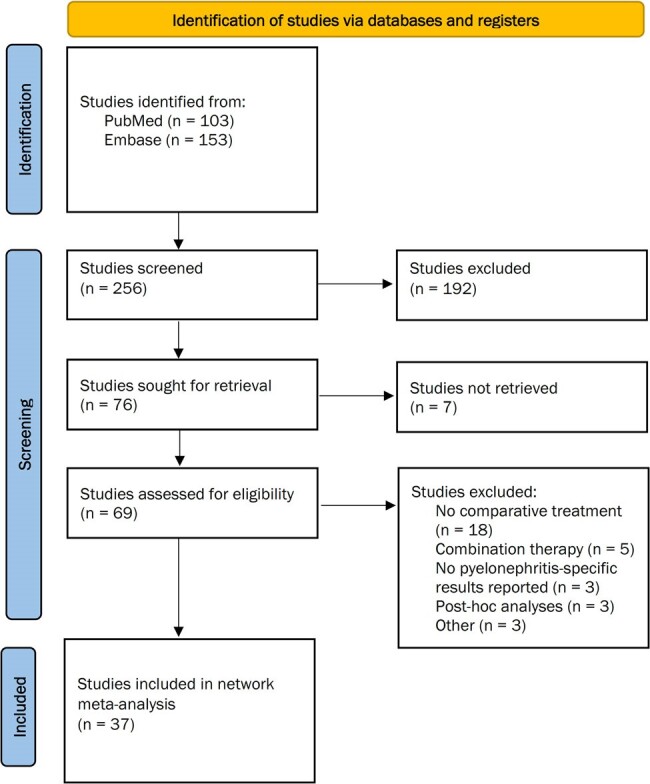
Flow diagram of study selection

**Methods:**

We conducted a systematic review of PubMed and Embase databases from inception to July 2023. We included randomized controlled trials (RCTs) involving adults (≥18 years) treated for acute pyelonephritis across various care settings. Our review focused on English-language articles examining intravenous (IV), oral, and IV-to-oral antibiotic treatments. Studies reporting outcomes specifically for pyelonephritis were eligible. We performed a frequentist network meta-analysis to evaluate clinical success, microbiological cure, and composite clinical or microbiological cure of different antibiotic therapies.

**Figure 2:**
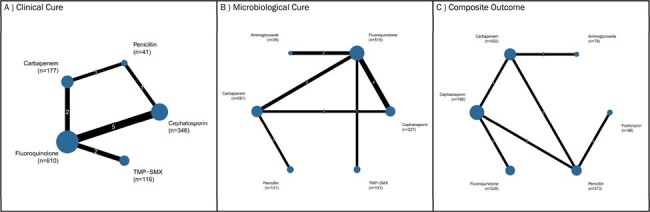
Network plot of RCTs evaluating A) clinical cure, B) microbiological cure, and C) composite outcome of clinical cure or microbiological cure

**Results:**

We included 37 RCTs (Figure 1). Due to data limitations, we assessed antibiotic classes rather than individual antibiotics (Figure 2). For clinical cure (n=1290, 11 RCTs), both fluoroquinolones (Odds Ratio [OR]: 3.71, 95% confidence interval [CI]: 1.30-10.55; P-score: 0.8215) and cephalosporins (OR: 3.45, 95% CI: 1.30-10.55; P-score: 0.7420) outperformed trimethoprim-sulfamethoxazole (TMP-SMX), with fluoroquinolones ranking highest in efficacy. For microbiological cure (n=1270, 11 RCTs), aminoglycosides (OR: 19.85, 95% CI: 1.62-243.61; P-score: 0.8373), fluoroquinolones (OR: 13.69, 95% CI: 1.73-108.03; P-score: 0.7785), cephalosporins (OR: 10.33, 95% CI: 1.20-88.73; P-score: 0.5395), and carbapenems (OR: 9.92, 95% CI: 1.14-86.61; P-score: 0.5394) were superior to TMP-SMX. Aminoglycosides ranked highest in efficacy. Regarding composite outcomes (n=2164, 6 RCTs), cephalosporins (P-score: 0.9898) and fluoroquinolones (0.7377) ranked highest in efficacy.

**Figure 3:**
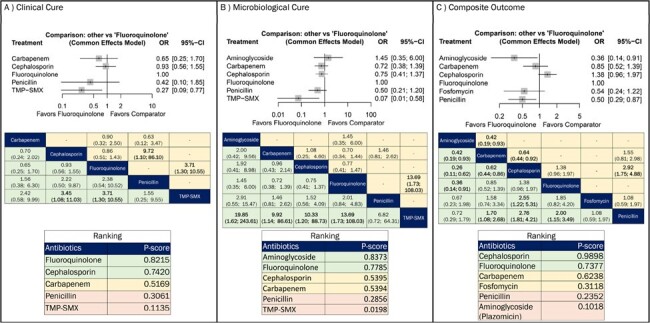
Efficacy of systemic antibiotics in A) clinical cure, B) microbiological cure, and C) composite outcome of clinical cure or microbiological cure in adult patients with acute pyelonephritis. League tables display the results [OR (95% CI)] of pairwise meta-analysis in the upper right half, when available, and the results of the network meta-analysis in the left lower half. CI, confidence interval; OR, odds ratio; TMP-SMX, trimethoprim-sulfamethoxazole.

**Conclusion:**

In adults with acute pyelonephritis, fluoroquinolones and cephalosporins demonstrate the highest efficacy probability, while TMP-SMX exhibits the lowest. Further studies are warranted to validate these findings.

**Disclosures:**

**All Authors**: No reported disclosures

